# Adhesion and invasion of gingival epithelial cells by *Porphyromonas gulae*

**DOI:** 10.1371/journal.pone.0213309

**Published:** 2019-03-14

**Authors:** Hiroaki Inaba, Ryota Nomura, Yukio Kato, Hiroki Takeuchi, Atsuo Amano, Fumitoshi Asai, Kazuhiko Nakano, Richard J. Lamont, Michiyo Matsumoto-Nakano

**Affiliations:** 1 Department of Pediatric Dentistry, Okayama University Graduate School of Medicine, Dentistry and Pharmaceutical Sciences, Okayama, Japan; 2 Department of Pediatric Dentistry, Osaka University Graduate School of Dentistry, Suita-Osaka, Japan; 3 Department of Veterinary Public Health II, School of Veterinary Medicine, Azabu University, Sagamihara, Kanagawa, Japan; 4 Department of Preventive Dentistry, Osaka University Graduate School of Dentistry, Suita-Osaka, Japan; 5 Department of Pharmacology, School of Veterinary Medicine, Azabu University, Sagamihara, Kanagawa, Japan; 6 Department of Oral Immunology and Infectious Diseases, School of Dentistry, University of Louisville, Louisville, KY, United States of America; University of Sheffield, UNITED KINGDOM

## Abstract

*Porphyromonas gulae*, an animal periodontal pathogen, possess fimbriae classified into three genotypes (A-C) based on the diversity of *fimA* genes encoding FimA. Accumulating evidence suggests that *P*. *gulae* strains with type C fimbriae are more virulent as compared to those with other types. The ability of these organisms to adhere to and invade gingival epithelial cells has yet to be examined. *P*. *gulae* showed the greatest levels of adhesion and invasion at a multiplicity of infection of 100 for 90 min. *P*. *gulae* type C and some type B strains invaded gingival epithelial cells at significantly greater levels than the other strains, at the same level of efficiency as *P*. *gingivalis* with type II fimbriae. Adhesion and invasion of gingival epithelial cells by *P*. *gulae* were inhibited by cytochalasin D and sodium azide, indicating the requirements of actin polymerization and energy metabolism for those activities. Invasion within gingival epithelial cells was blocked by staurosporine, whereas those inhibitors showed little effects on adhesion, while nocodazole and cycloheximide had negligible effects on either adhesion or invasion. *P*. *gulae* proteases were found to be essential for adhesion and invasion of gingival epithelial cells, while its DNA and RNA, and protein synthesis were unnecessary for those activities. Additionally, α5β1 integrin antibodies significantly inhibited adhesion and invasion by *P*. *gulae*. This is the first report to characterize *P*. *gulae* adhesion and invasion of human gingival epithelial cells.

## Introduction

*Porphyromonas gulae*, previously known as the animal biotype of the human periodontal pathogen *P*. *gingivalis*, is a black-pigmented rod-shaped organism, with asaccharolytic, anaerobic, non-motile, non-spore-forming, and gram-negative characteristics [1]. *P*. *gulae* organisms have been isolated from the gingival sulcus of various animal species, including bear, brushtail possum, dog, cat, coyote, kangaroo, monkey, ovine, wallaby, and wolf [1–3]. Furthermore, this bacterium has been detected in significantly higher levels in the gingival sulcus of dogs with periodontitis as compared to healthy specimens [4, 5]. Recent studies have reported that *P*. *gulae* was detected in human gingival tissues from healthy and diseased site [6]. In addition, *P*. *gulae* infection reportedly induced inflammatory responses and diminished cellular motility in human cell lines [7].

*P*. *gulae* possesses surface fimbrial appendages composed of a 41 kDa subunit protein (fimbrillin; FimA) [8]. The *P*. *gulae fimA* genes encoding FimA have been classified into types A, B, and C based on their nucleotide sequences [9], and recent studies have shown a link between *P*. *gulae fimA* type and periodontal pathogenicity [9, 10]. A polymerase chain reaction (PCR) assay using *fimA* type-specific primers has been developed to differentiate *fimA* types among organisms detected in oral swab specimens obtained from dogs with periodontitis, with a majority of such animals found to harbor those with type B and/or C *fimA* [9]. In addition, *P*. *gulae* with type C fimbriae has been shown to be have greater levels of virulence towards mouse and human oral epithelial cells as compared to other types, suggesting an association of type C fimbriae with elevated risk for developing periodontitis [9].

Bacterial adherence to host cell surfaces is often the essential first stage in successful establishment of infection [11, 12]. Following adherence, bacterial pathogens colonize the tissue and can enter into target cells, leading to bacterial disease [12]. Furthermore, cellular invasion is considered to be an important virulence factor, as it provides an opportunity for escape from the host immune system, thus contributing to tissue damage [13]. Fimbriae of various species are known to play an important role in bacterial adherence to cell surfaces [11], as they are able to recognize several different membrane cellular receptors, such as integrins, cadherins, selectins, and carcinoembryonic antigen-related adhesion molecules, which are involved in mediating bacterial invasion [12]. Various pathogens, such as the *Salmonella*, *Shigella*, *Yersinia*, and *Listeria* genera, adhere to integrin α5β1 and trigger actin cytoskeleton rearrangements, leading to cellular invasion [12]. In addition, the interaction with integrin α5β1 by *P*. *gingivalis* fimbriae is involved in bacterial adhesion and invasion [14, 15]. On the other hand, *P*. *gulae* adhesion and invasion characteristics remain largely unknown. The present study is the first to elucidate the process of invasion of human gingival epithelial cells by *P*. *gulae*, in which we examined optimal conditions for adhesion and invasion, and also analyzed inhibitors of bacterial and epithelial cell functions.

## Materials and methods

### Bacterial and cell cultures

*P*. *gulae* strains ATCC 51700 (type A), D040 (type B), D044 (type B), D049 (type C), D066 (type A), and ST9-1 (type C), and *P*. *gingivalis* strains ATCC33277 (type I) and OMZ314 (type II) were selected from our culture collections. Bacterial cells were grown in Trypticase soy broth supplemented with yeast extract (1 mg/ml), menadione (1 μg/ml), and hemin (5 μg/ml), as described previously [16]. Ca9-22 (originated from human gingival epithelia) and SAS (originated from human tongue) cells were obtained from the Japanese Collection of Research Bioresources (Tokyo, Japan), and cultured in Dulbecco’s modified Eagle’s medium (DMEM) (Wako, Osaka, Japan) supplemented with 10% fetal bovine serum (FBS) at 37°C in 5% CO_2_.

### Antibiotic protection adhesion and invasion assay

Assays of bacterial adherence and invasion were performed using methods previously described [17]. Briefly, *P*. *gulae* strains were harvested, washed, and resuspended in DMEM without antibiotics. Ca9-22 cells were infected with bacteria at an MOI ranging from 1–1000 for 0–120 min, and washed with phosphate-buffered saline (PBS). For determining total adhesion and invasion levels, cells were lysed with sterile distilled water for 15 minutes, then dilutions of the lysate were plated and cultured anaerobically on blood agar supplemented with hemin and menadione to determine the number of colony forming units. For invasion assay, extracellular bacteria were killed with ampicillin (200 μg/ml) and gentamicin (300 μg/ml) for 1 h. Those concentrations of antibiotics were sufficient to completely kill 10^9^ bacteria per ml in 1 h (S1 Fig).

### Chemicals

Cycloheximide, nocodazole, sodium azide, chloramphenicol, rifampin, nalidixic acid, and a cocktail of protease inhibitors cocktail were purchased from Wako. Cytochalasin D was purchased from Focus Biomolecules (Plymouth Meeting, PA) and staurosporine from Cayman Chemical Company (Ann Arbor, MI). The solvents used and final concentrations were as follows: cycloheximide, 100 μg/ml in dimethyl sulfoxide (DMSO); nocodazole, 10 μg/ml in DMSO; sodium azide, 50 mM in PBS; chloramphenicol, 5 μg/ml in ethanol; rifampin, 0.25 mg/ml in DMSO; nalidixic acid, 5 μg/ml in 1N NaOH; protease inhibitor cocktail containing aminoethyl benzylsulfonyl fluoride (AEBSF), 100 mM in aprotinin, 80 μM in DMSO; E-64, 1.5 mM in DMSO, leupeptin, 2 mM, bestatin, 5 mM, pepstatin, 1 mM; cytochalasin D, 1 μg/ml in DMSO; and staurosporine, 1 μM in ethyl acetate. All chemicals and solvents were tested at a concentration used for possible adverse effects in Ca9-22 cells, as compared with cells without the inhibitor, by examining the morphology of the cells and cell viability with MTT assay (S2 Fig). Moreover, all chemicals and solvents were tested at the appropriate concentrations and found to produce no reduction in *P*. *gulae* growth (S3 Fig).

### Western immunoblotting

Ca9-22 and SAS cells were solubilized in cell lysis/extraction reagent (Sigma-Aldrich, St. Louis, MO) containing a protease inhibitor cocktail (Wako). Immunoblotting was performed as previously described [16]. Blots were probed at 4°C overnight with the following primary antibodies: anti-integrin α5, 1:1000; anti-integrin β1, 1:1000; anti-integrin β3, 1:1000 (GeneTex, Irvine, CA); anti-integrin αV, 1:1000 (BD Transduction Laboratories, San Diego, CA). Proteins were detected using the ECL Western Blotting detection reagents (Amersham Pharmacia Biotech, Little Chalfont, UK). Blots were stripped and probed with anti-β-actin antibody (Cell Signaling Technology, Beverly, MA) as a loading control.

### Inhibitors of bacterial and gingival epithelial cell function

The effects of a variety of inhibitors of prokaryotic and eukaryotic cell functions on *P*. *gulae* invasion of Ca9-22 cells were investigated. Cycloheximide was preincubated with Ca9-22 cells for 4 h prior to bacterial infection and present during the assay. Epithelial cells were preincubated with cytochalasin D and staurosporine for 30 min prior to infection of the bacteria, and remained present throughout the invasion assay. Ca9-22 cells were preincubated with nocodazole for 1 h on ice and then at 37°C for 30 min prior to reacting with the bacteria, which remained present during the invasion assay. Sodium azide was preincubated with Ca9-22 cells for 4 h and then that was removed by washing three times in DMEM prior to bacterial infection. Sodium azide were also preincubated with *P*. *gingivalis* or *P*. *gulae* for 4 h, which was then removed by washing prior to the assay. Bacteria were preincubated with chloramphenicol, rifampin, and nalidixic acid for 4 h before reaction with Ca9-22 cells, and those chemicals were present during the assay. Protease inhibitors were preincubated with *P*. *gingivalis* or *P*. *gulae* for 30 min prior to the assay.

### Electron microscopy

SEM and TEM were performed to observe Ca9-22 cells infected with bacteria at an MOI of 100 for 90 min. For TEM, infected and control Ca9-22 cells were washed with PBS, then fixed in 2% paraformaldehyde and 2% glutaraldehyde in 0.1 M cacodylate buffer (pH 7.4) at 4°C, then dehydrated in graded ethanol and embedded in resin (Quetol-812; Nisshin EM Co., Tokyo, Japan). Ultrathin sections were stained with 2% uranyl acetate and observed using TEM (JEM-1400Puls; JEOL Ltd., Tokyo, Japan) at an accelerating voltage of 80 kV. Digital micrographs were acquired directly using a CCD camera system (EM-14830RUBY2; JEOL Ltd., Tokyo, Japan). For SEM, infected and control Ca9-22 cells were washed with PBS, then fixed in 2% glutaraldehyde in 0.1 M cacodylate buffer (pH 7.4) at 4°C overnight. Additionally, specimens were fixed in 1% tannic acid in 0.1 M cacodylate buffer (pH 7.4) and stained for 1 h in 2% osmium tetroxide. Following dehydration in a graded series of alcohols and drying, specimens were coated with a thin layer using an osmium plasma coater (NL-OPC80NS; Nippon Laser & Electronics Laboratory, Nagoya, Japan). Examinations were performed using SEM (JSM-6340F; JEOL Ltd., Tokyo, Japan) at an accelerating voltage of 5.0 kV.

### Normal human serum and plasma treatments

Single donor human serum and plasma samples were used (Innovative Research, Novi, MI). Following series of dilutions, serum and plasma were incubated with bacterial suspensions at 37°C for 1 h prior to reacting with Ca9-22 cells. Neither caused agglutination of bacteria and remained present throughout the invasion assay.

## Results

### Adherence and invasion characteristics

**Fig 1:**
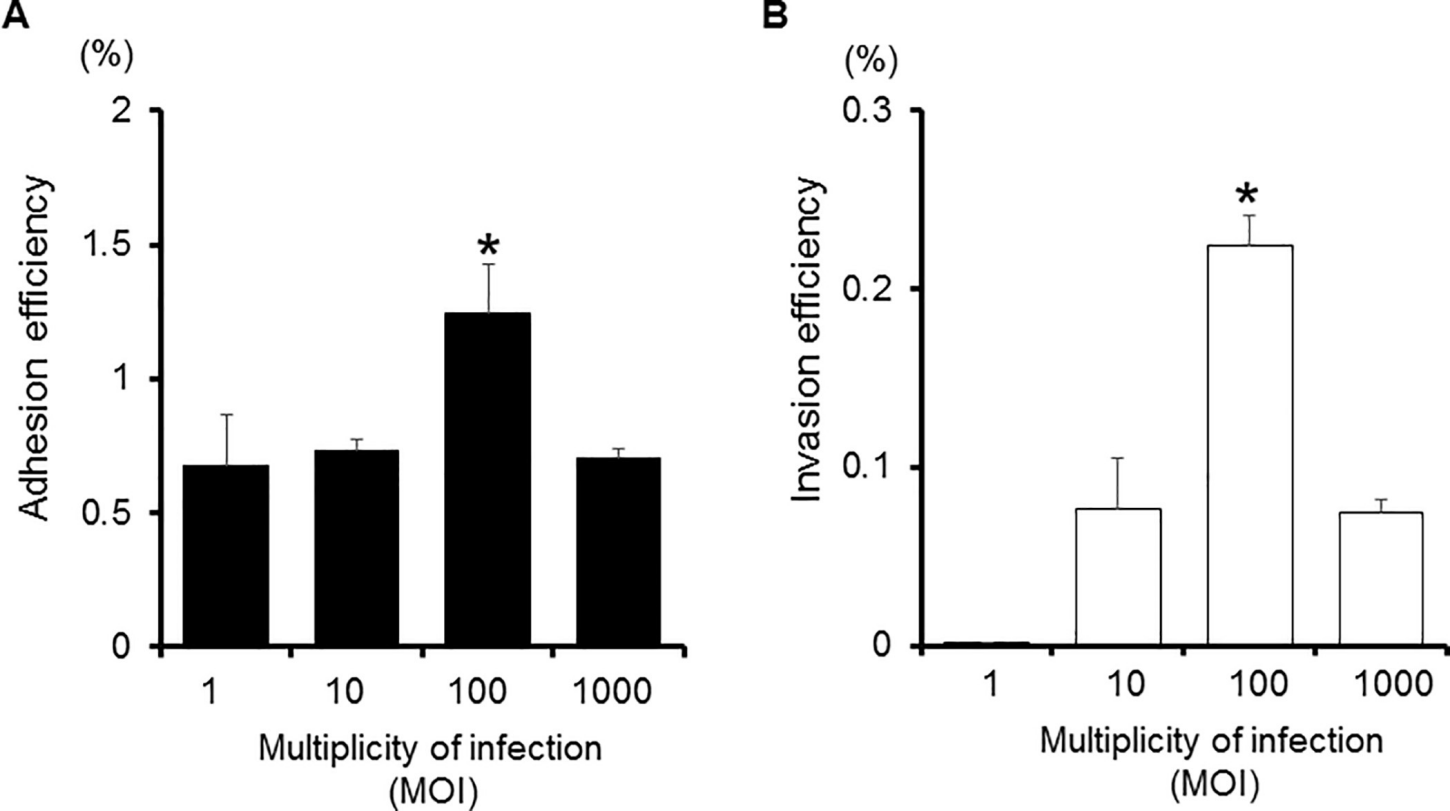
Effects of MOI on adhesion and invasion of Ca9-22 cells by *P*. *gulae* D049. Antibiotic protection invasion assays with *P*. *gulae* D049. Ca9-22 cells were infected with *P*. *gulae* at an MOI of 1, 10, 100, or 1000 for 90 min. The numbers of adherent and/or intracellular bacteria were determined by counting viable cell lysates, and are expressed as percentage of input bacterial cell number. Values are shown as the mean ± SD of three independent experiments and were analyzed with a t test. *P <0.01 (Student’s t test), as compared with infected cells (MOI 1, 10, and/or 1000). A; Adhesion efficiency, B; Invasion efficiency.

**Fig 2:**
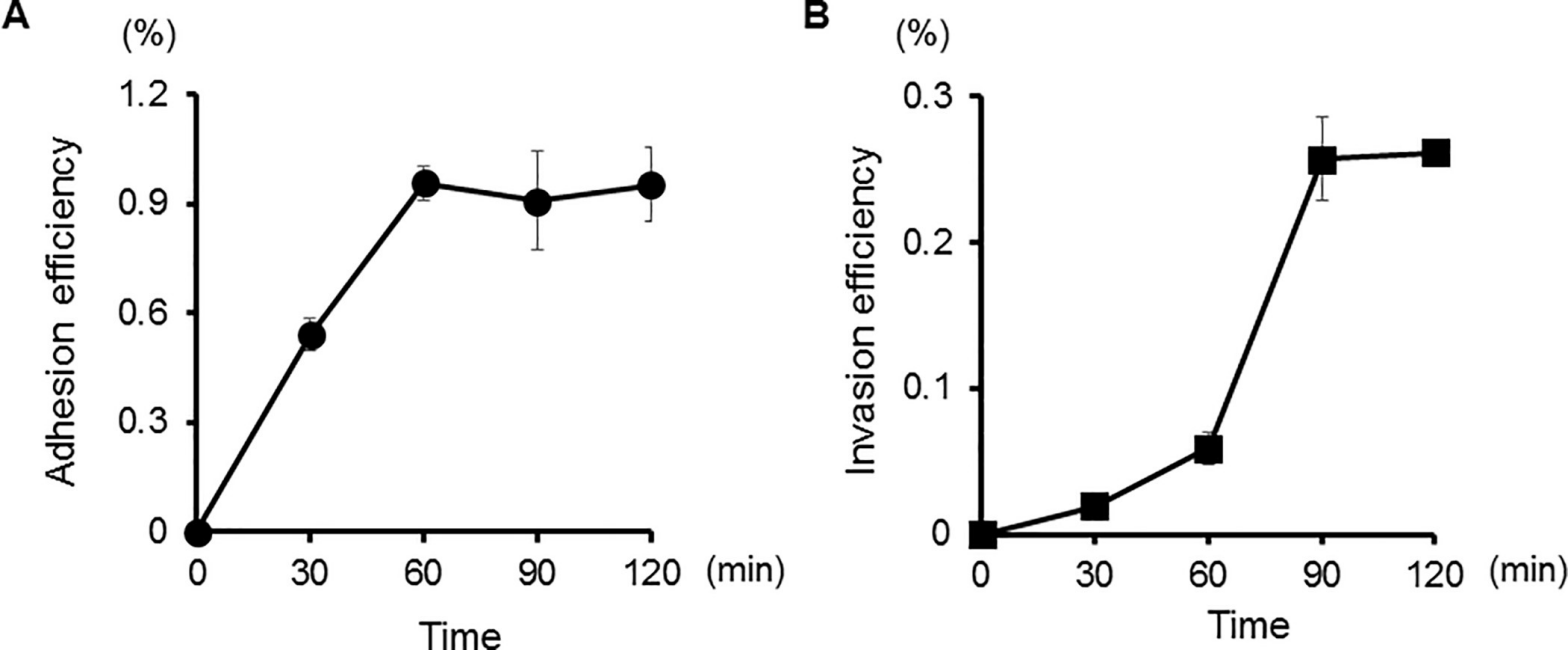
Time course for adhesion and invasion of Ca9-22 cells by *P*. *gulae* D049. Ca9-22 cells were infected with *P*. *gulae* at an MOI of 100 for the indicated times. Numbers of adherent and/or intracellular bacteria were determined by counting viable cell lysates, and are expressed as percentage of input bacterial cell number. Values are shown as the mean ± SD of three independent experiments. A; Adhesion efficiency, B; Invasion efficiency.

The ability of *P*. *gulae* D049 to adhere to and invade cultured Ca9-22 cells was determined using an antibiotic protection assay. Examination using multiplicity of infection (MOI) values ranging from 1 to 1000 revealed the most efficient levels of adhesion and invasion occurred at an MOI of 100 (Fig 1A and 1B), which was utilized in all subsequent experiments. Fig 2 shows a typical time course for adhesion and invasion of Ca9-22 cells by *P*. *gulae*. Adhesion efficiency increased with incubation time up to 60 min (Fig 2A), while invasion efficiency increased with that up to 90 min (Fig 2B). Therefore, in subsequent experiments, bacteria were incubated with Ca9-22 cells for 90 min.

### Adhesion and invasion of *P*. *gulae* with different *fimA* genotypes

**Fig 3:**
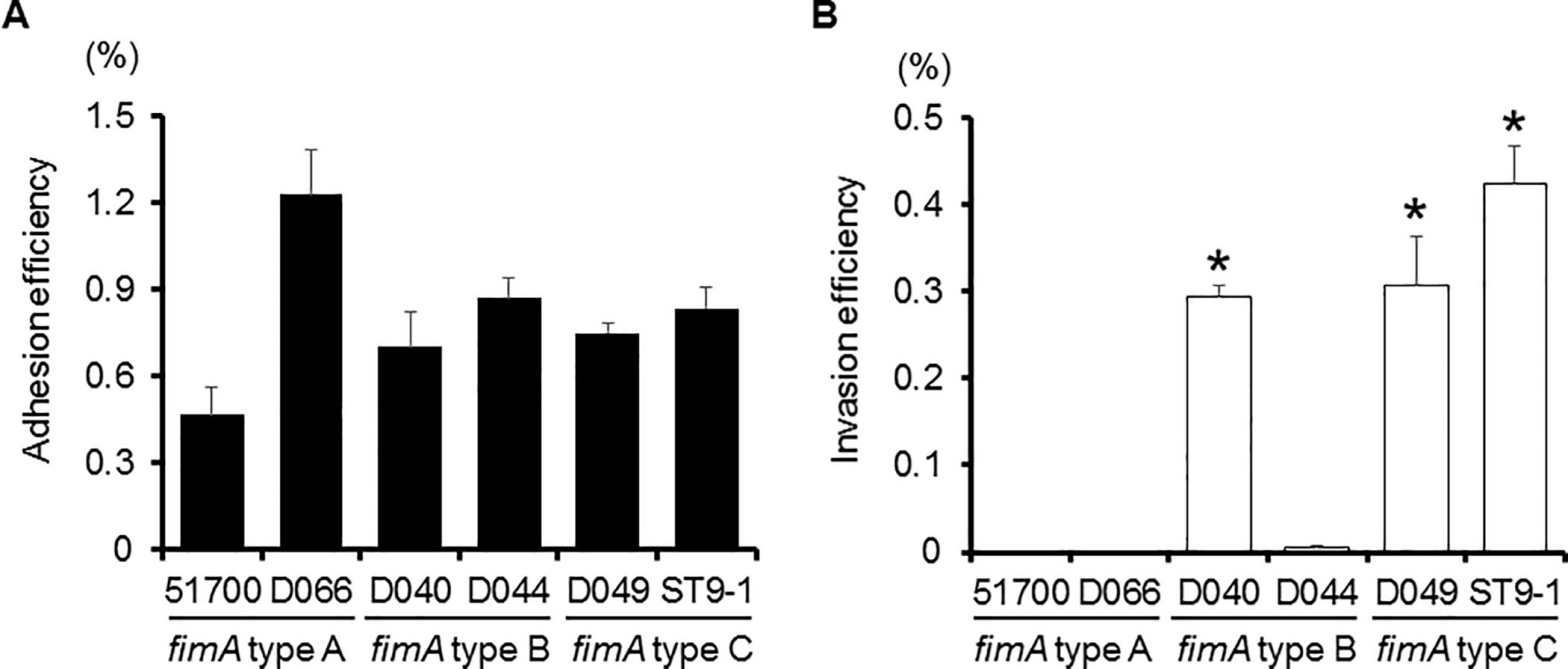
**Adhesion/invasion of Ca9-22 cells by *P*. *gulae* strains with distinct types of fimbriae (types A, B and C).** Antibiotic protection invasion assays of *P*. *gulae* strains. Ca9-22 cells were infected with the bacteria at an MOI of 100 for 90 min. The numbers of adherent and/or intracellular bacteria were determined by counting viable cell lysates and are expressed as percentage of input bacterial cell number. Values are shown as the mean ± SD of three independent experiments. A; Adhesion efficiency, B; Invasion efficiency. *P <0.01 (Student’s t test), as compared with infected cells (51700, D066 and/or D044 strains).

**Fig 4:**
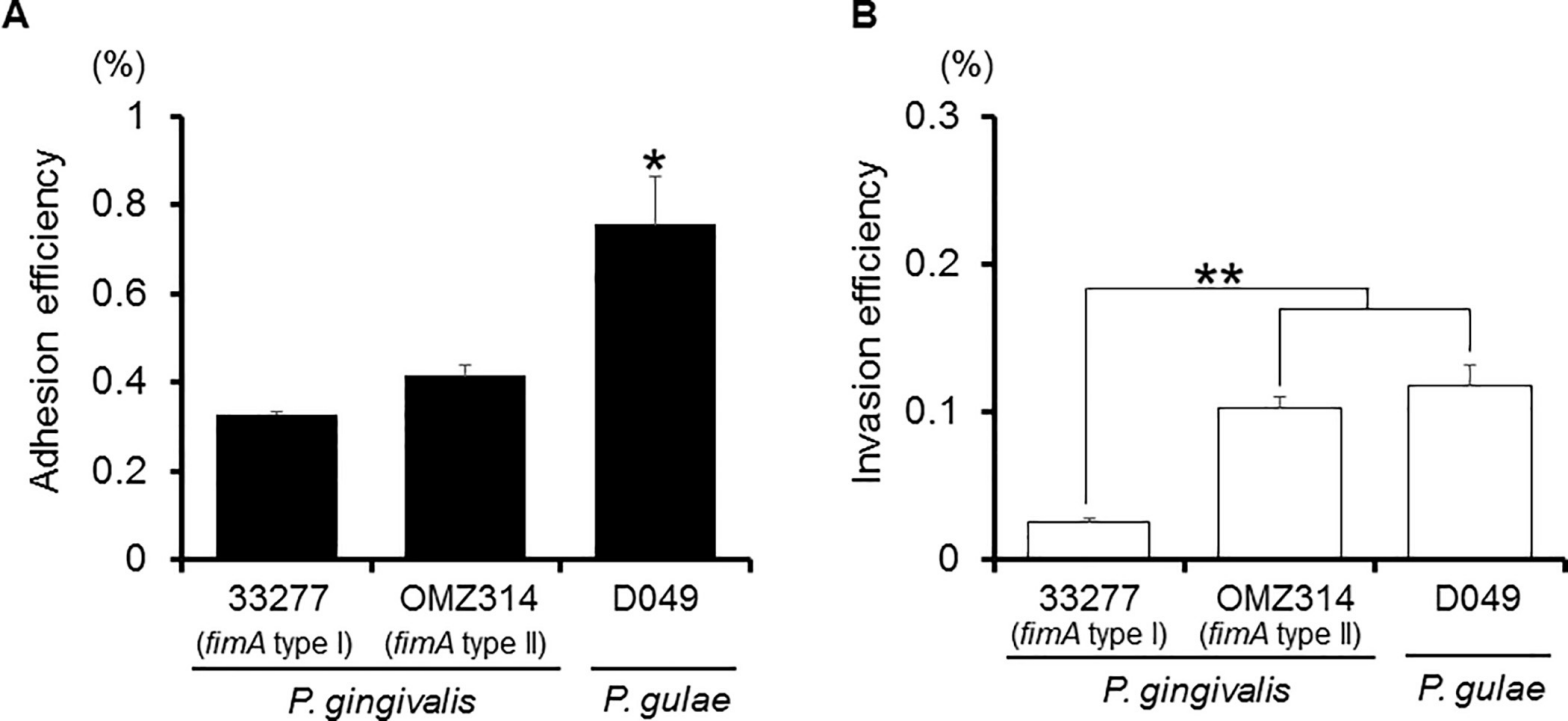
Adhesion/invasion of Ca9-22 cells by *P*. *gulae* D049 as compared with *P*. *gingivalis* strains. Antibiotic protection invasion assay of *P*. *gulae* D049, and *P*. *gingivalis* 33277 and OMZ314. Ca9-22 cells were infected with bacteria at an MOI of 100 for 90 min. The numbers of adherent and/or intracellular bacteria were determined by counting viable cell lysates and are expressed as percentage of input bacterial cell number. Values are shown as the mean ± SD of three independent experiments and were analyzed with a t test. *P <0.05, and **P <0.01 (Student’s t test) as compared with *P*. *gingivalis* 33277 and/or OMZ314. A; Adhesion efficiency, B; Invasion efficiency.

The efficiency of adhesion and invasion by bacteria can be dependent to some extent on the clonal diversity of fimbriae. *P*. *gingivalis*, a periodontal pathogen, is classified into six types (I to V and Ib) based on the *fimA* genes encoding the FimA fimbrial subunit [18]. *P*. *gingivalis* with type II fimbriae have been shown to adhere to and invade epithelial cells at significantly greater levels than strains with other types [14]. In addition, pathogenic heterogeneity exists among *P*. *gingivalis* clones with type II fimbriae [19]. *P*. *gulae fimA* genes are classified into three variants (types A, B and C) [6], and to examine whether the adhesion and invasion efficiency of *P*. *gulae* is dependent on *fimA* type, we used strains with distinct types. We found that adhesion by *P*. *gulae* D066 (type A) was significantly greater as compared to the other types, while the adhesion abilities did not differ significantly among the other examined strains (Fig 3A). As for invasion, that of type C strains and the D040 strain (*fimA* type B) was significantly greater than the others (Fig 3B), whereas the invasion abilities of type A strains and D044 (type B) were negligible. We next examined the adhesion and invasive efficiency of *P*. *gulae* as compared with various *P*. *gingivalis* strains. *P*. *gulae* D049 (type C) adhesion was significantly greater than that of the *P*. *gingivalis* strains (Fig 4A). In addition, *P*. *gulae* D049 (type C) and *P*. *gingivalis* OMZ314 (type II) showed invasion at a higher level of efficiency than *P*. *gingivalis* ATCC 33277 (type I) (Fig 4B).

### Effects of metabolic inhibitors on adhesion and invasion

The effects of selected inhibitors of eukaryotic and prokaryotic cell functions were examined regarding adhesion to and invasion of Ca9-22 cells by *P*. *gulae* D049 (Table 1). Cytochalasin D, an inhibitor of actin polymerization, significantly decreased adhesion by 23.86% and invasion by 56.47%, whereas nocodazole, a microtubule depolymerizing agent, showed negligible effects. Additionally, staurosporine, an inhibitor of protein kinase C, reduced invasion by 41.80%, but showed scant effect on adhesion, while cycloheximide, a eukaryotic protein synthesis inhibitor, had negligible effects on either adhesion or invasion. Interruption of the energy metabolism of *P*. *gulae* as well as of the epithelial cells also reduced bacterial adhesion and invasion, suggesting the importance of energy metabolism. Bacterial DNA and RNA synthesis, and protein synthesis appear to be required for adhesion and invasion, since chloramphenicol, rifampin, nalidixic acid reduced *P*. *gulae* adhesion and invasion of Ca9-22 cells.

**Table 1:** Effects of metabolic inhibitors on *P*. *gulae* D049 adhesion and invasion of Ca9-22 cells.

Inhibitor	Target	Inhibition of adhesion (%)	Inhibition of invasion (%)
Cytochalasin D	Cell (actin)	23.86 ± 2.76	56.47 ± 1.20
Nocodazole	Cell (microtubule)	7.61 ± 3.51	4.12 ± 2.26
Staurosporine	Cell (protein kinase C)	3.16 ± 2.27	41.80 ± 4.97
Cycloheximide	Cell (protein synthesis)	4.71 ± 2.91	6.07 ± 2.24
Sodium azide	Cell (energy metabolism)	11.4 ± 1.75	61.17 ± 4.70
Sodium azide	*P*. *gulae* (energy metabolism)	11.11 ± 4.51	66.00 ± 5.16
Chloramphenicol	*P*. *gulae* (protein synthesis)	54.04 ± 2.41	52.23 ± 2.31
Rifampin	*P*. *gulae* (RNA synthesis)	50.02 ± 2.65	54.58 ± 0.80
Nalidixic acid	*P*. *gulae* (DNA synthesis)	50.98 ± 2.11	51.86 ± 1.89
Protease inhibitors	*P*. *gulae* (proteases)	99.65 ± 0.50	100.00

### Effects of protease inhibitor on adhesion and invasion

A cocktail of protease inhibitors containing AEBSF, aprotinin, E-64, leupeptin, bestatin, and pepstatin, at concentrations predetermined to not be lethal to Ca9-22 cells or *P*. *gulae*, abrogated adhesion by 99.65% and invasion by 100% (Table 1).

### Effects of human serum and plasma on adhesion and invasion

The constituents of gingival crevicular fiuid (GCF) are derived from plasma and periodontal tissues, and include serum transudate and inflammatory exudate [20, 21]. Thus, human serum and plasma were used as surrogates for GCF to investigate possible modulation of adhesion and invasion. Human serum abrogated both adhesion and invasion by *P*. *gulae* D049 (Table 2), whereas human plasma did not show either of those effects (Table 3).

**Table 2:** Effects of human serum on *P*. *gulae* adhesion and invasion of Ca9-22 cells.

(%)	Control ^a^	In the presence of human plasma diluted :		
		1 : 10	1 : 100	1 : 1000
Adhesion	1.11 ± 0.06	**1.01 ± 0.15	**1.01 ± 0.14	*1.04 ± 0.12
Invasion	0.41 ± 0.03	**0.35 ± 0.05	**0.39 ± 0.04	0.40 ± 0.03

^a^ Absence of human serum

*p<0.05

** *p*<0.01

**Table 3:** Effects of human plasma on *P*. *gulae* adhesion and invasion of Ca9-22 cells.

(%)	Control ^a^	In the presence of human plasma diluted :		
		1 : 10	1 : 100	1 : 1000
Adhesion	1.11 ± 0.06	1.01 ± 0.15	1.01 ± 0.14	1.04 ± 0.12
Invasion	0.41 ± 0.03	0.35 ± 0.05	0.39 ± 0.04	0.40 ± 0.03

^a^ Absence of human plasma

### Involvement of integrin α5β1 with adhesion and invasion

**Fig 5:**
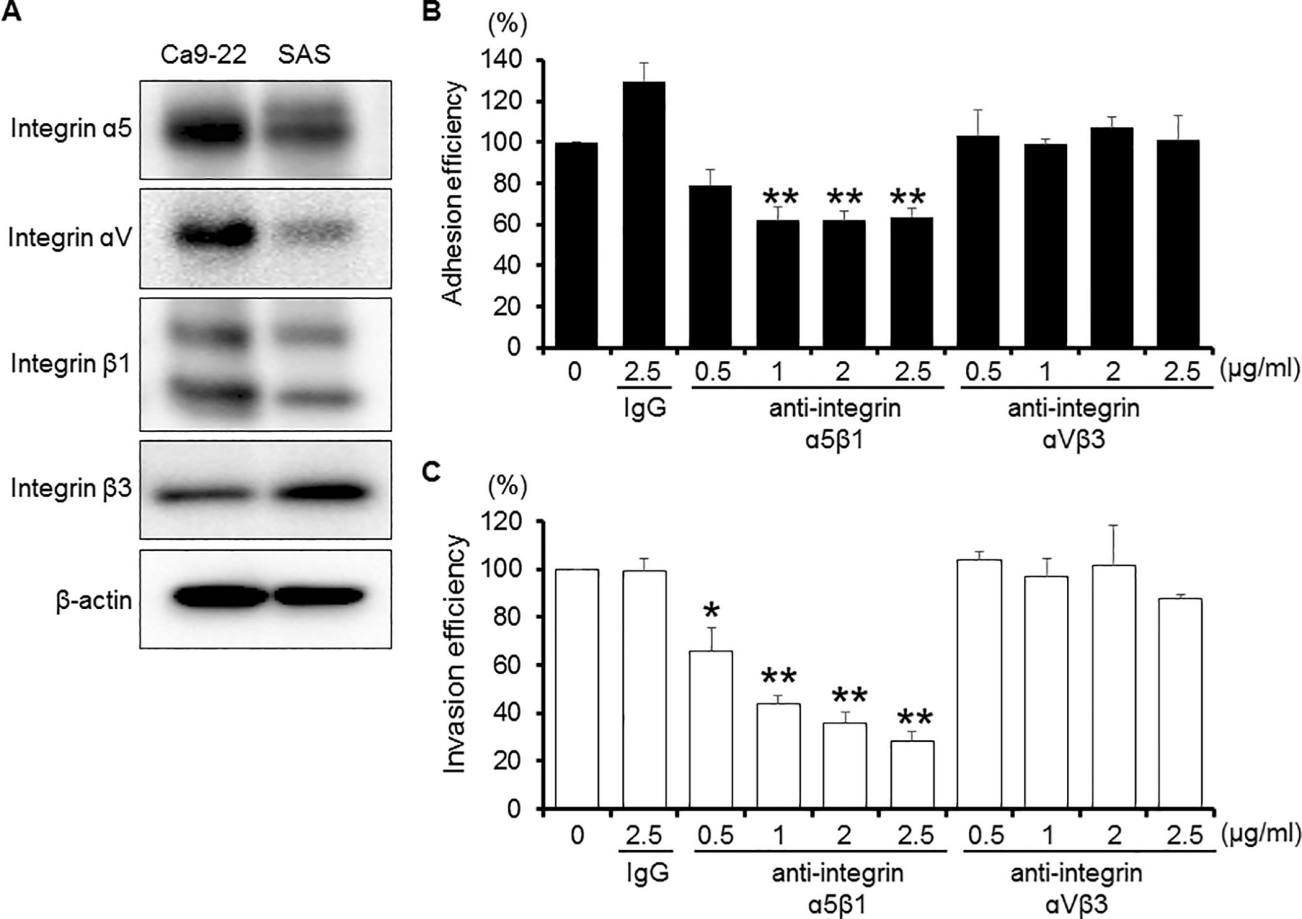
Inhibition of *P*. *gulae* D049 adhesion and invasion by anti-integrin α5β1 antibody. A: Expressions of integrins in Ca9-22 cells. Cells were lysed and immunoblotted with anti-integrin α5, αV, β1, β3, or β-actin antibodies. SAS cells were used as a positive control. B: Ca9-22 cells were infected with *P*. *gulae* D049 at an MOI of 100 for 90 min with/without anti-integrin antibodies. The numbers of adherent bacteria were determined by counting viable cell lysates and are expressed as percentage of input bacterial cell number. Values are shown as the mean ± SD of three independent experiments and were analyzed with a t test. **P <0.01 (Student’s t test) as compared with 0 (no anti-integrin antibodies or IgG). IgG, immunoglobulin G. C: Ca9-22 cells were infected with *P*. *gulae* D049 at an MOI of 100 for 90 min with/without anti-integrin antibodies. The numbers of intracellular bacteria were determined by counting viable cell lysates and are expressed as percentage of input bacterial cell number. Values are shown as the mean ± SD of three independent experiments and were analyzed with a t test. *and **, *P* < 0.05 and *P* <0.01 (Student’s t test) as compared with 0 (no anti-integrin antibodies or IgG). IgG, immunoglobulin G.

The integrin family of integral membrane proteins function as receptors of several types of pathogens for adhesion and cell entry. Notably, a variety of pathogens have been reported to exploit integrin α5β1 or αVβ3 for adhesion to host cell surfaces, resulting in invasion [12, 22]. Therefore, we examined the expression profiles of the integrins α5, αV, β1, and β3, and each was confirmed to be expressed on both Ca9-22 cells and SAS cells (positive control) (Fig 5A). Next, we examined whether the adhesion and invasion process of *P*. *gulae* are mediated by integrins α5β1 or αVβ3. Anti-integrin α5β1 antibodies were found to inhibit the adherence of *P*. *gulae* by up to 46.2% as well as invasion by up to 71.5% (Fig 5B and 5C). In contrast, control IgG and integrin αVβ3 antibodies did not exhibit such effects. These findings suggest that integrin α5β1 is involved in adhesion to and invasion of Ca9-22 cells by *P*. *gulae*.

### Electron microscopy

**Fig 6:**
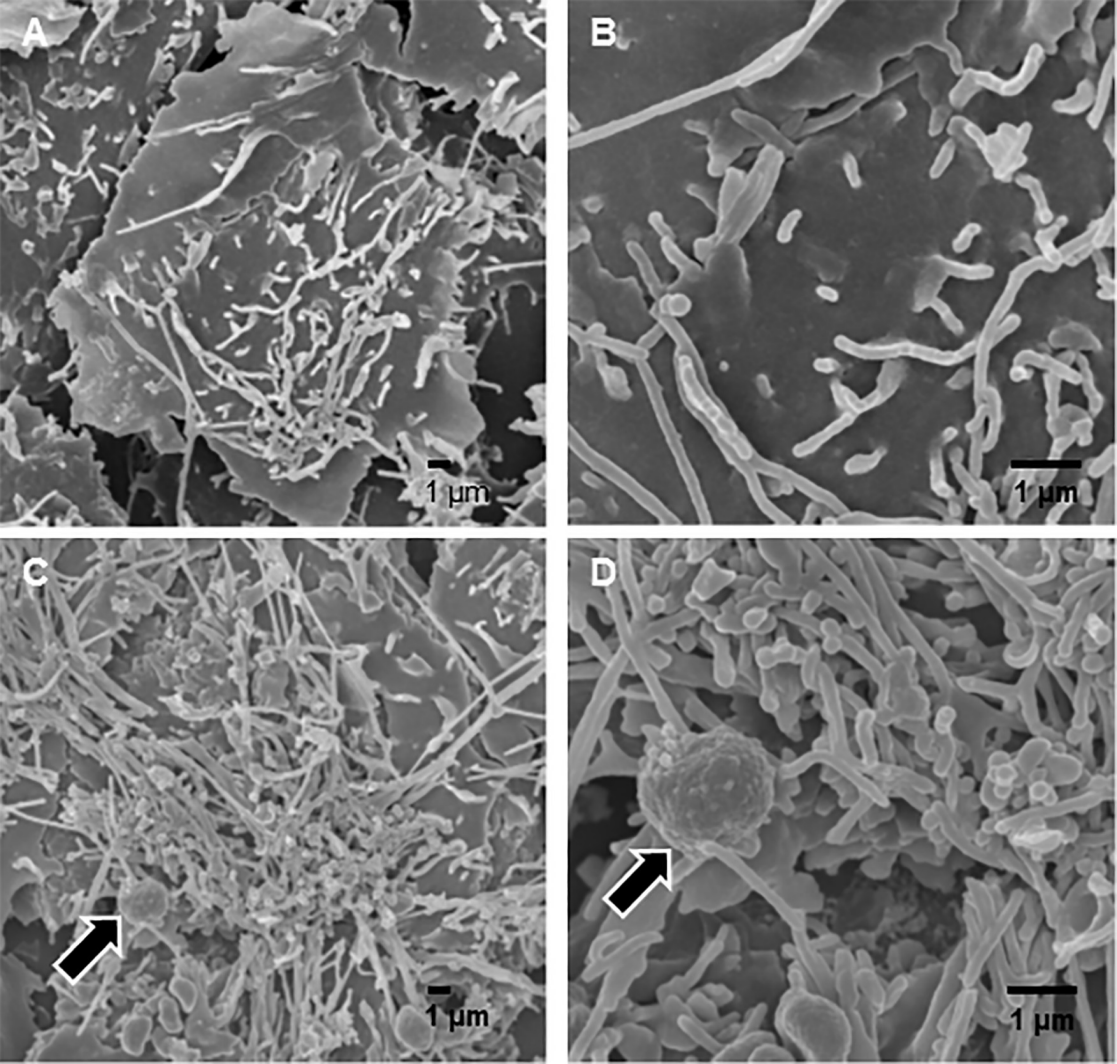
Scanning electron microscopy of Ca9-22 cells infected with *P*. *gulae* D049. SEM examinations of Ca9-22 cells infected with/without *P*. *gulae* D049 at an MOI of 100 for 90 min. Bar markers represent 1 μm. A, Uninfected Ca9-22 cells (lower magnification). B: Uninfected Ca9-22 cells (higher magnification). C: Lower magnification showing engulfment of *P*. *gulae* by cellular protrusions during localized adhesion step of infection (arrows). D: Higher magnification showing cellular protrusions engulfing *P*. *gulae* (arrows).

**Fig 7:**
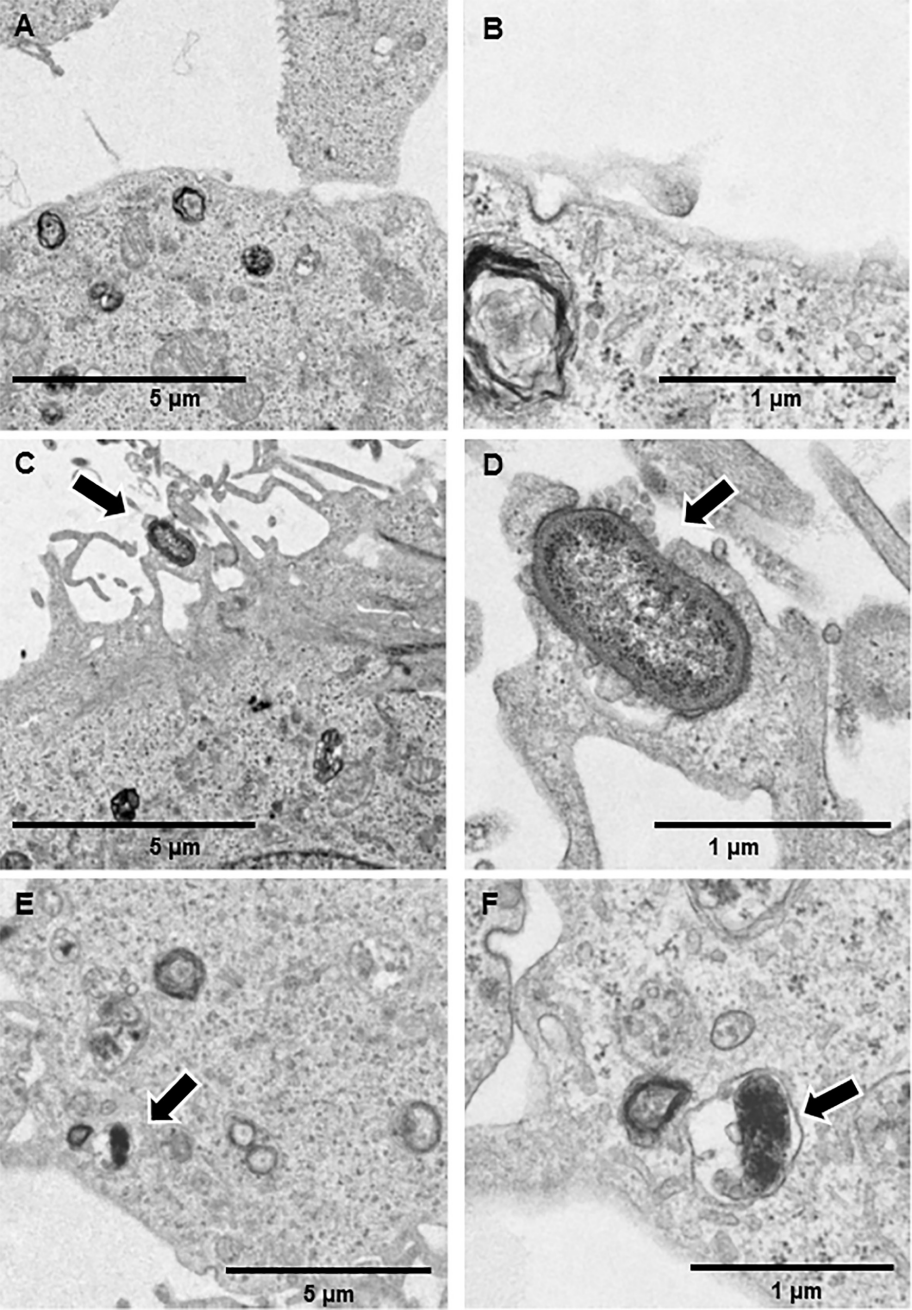
Transmission electron microscopy of Ca9-22 cells infected with *P*. *gulae* D049. TEM examinations of Ca9-22 infected with/without *P*. *gulae* D049 at an MOI of 100 for 90 min. Bar markers represent 5 μm (A, C and E) or 1 μm (B, D and F). A: Uninfected Ca9-22 cells (lower magnification). B: Uninfected Ca9-22 cells (higher magnification). C: Lower magnification showing adherent *P*. *gulae* and subsequent engulfment by cellular protrusions (arrows). D: Higher magnification showing adherent *P*. *gulae* and subsequent engulfment by cellular protrusions (arrows). E: Lower magnification showing intracellular *P*. *gulae* in vacuole (arrows). F: Higher magnification showing intracellular *P*. *gulae* in vacuole (arrows).

Finally, we examined the interaction between *P*. *gulae* with Ca9-22 cells using electron microscopy. Scanning electron microscopy (SEM) analysis showed that *P*. *gulae* induced significant formation of long microvilli surrounding the bacteria (Fig 6), while transmission electron microscopy (TEM) analysis showed that *P*. *gulae* organisms are engulfed by long microvilli, leading to formation of vacuoles containing *P*. *gulae* (Fig 7). Our results suggest that these long microvilli are associated with cellular invasion by *P*. *gulae*.

## Discussion

The fimbriae-mediated adhesion and invasion abilities of periodontal organisms is highly relevant to pathogenicity [23]. *P*. *gingivalis* fimbriae mediate bacterial interactions for host tissue invasion and are thus likely involved in development of periodontitis [24]. *P*. *gingivalis* organisms with *fimA* type II fimbriae were reported to be more frequently detected in deeper pockets (≥8 mm) and significantly more predominant in periodontitis patients [25]. In addition, *P*. *gingivalis* with type II fimbriae showed significantly greater levels of bacterial adhesion and invasion as compared to other strains with other fimbriae types [14], while they were also found to exhibit strong cellular cytotoxicity [26, 27]. These reports indicate that the diversity of fimbriae exerts an influence on bacterial adhesion and invasion, as well as cytotoxicity, pathogenicity and development of disease. In the present study, *P*. *gulae* with type C fimbriae consistently showed greater invasion as compared to the other examined strains (Fig 3B), which was equivalent to that of *P*. *gingivalis* with type II fimbriae (Fig 4B). Collectively, these results suggest that type C and some type B strains have a strong relationship to bacterial invasion of host cells, with *P*. *gulae* possessing type B and type C fimbriae potentially playing a crucial role in development of periodontitis. On the other hand, there were no significant differences regarding the abilities of each type of fimbriae to adhere to human gingival cells (Fig 3A). Together, our findings suggest that type B and C fimbriae may possess a capability to induce epithelial cell cytoskeletal rearrangement, while type A fimbriae have little or none the ability.

Optimal invasion efficiency of *P*. *gulae* occurred at an MOI 100 (Fig 1B), which was similar to that of several other invasive pathogens previously reported, such as *Bartonella henselae* [28], *Neisseria gonorrhoeae* [29], *P*. *gingivalis* [13], and *Staphylococcus aureus* [30]. On the other hand, the optimal MOI for a few strains, such as *Mycobacterium tuberculosis* [31], *Serratia marcescens* [32], and *Yersinia pestis* [33], were lower than MOI100, while that of others, such as *S*. *epidermidis* [34], was higher than MOI100. While the results indicate that the ability of *P*. *gulae* strains to adhere to and invade Ca9-22 cells is very low compared to *B*. *henselae* [28], *N*. *gonorrhoeae* [29], *S*. *aureus* [30], and *S*. *epidermidis* [34], other studies with Ca9-22 cell lines have reported that the invasion efficiency of the *P*. *gingivalis* W83 strain were less than 0.6% at MOI 100 [35, 36]. However, this strain invaded human aorta endothelial cells approximately threefold higher than Ca9-22 cells [35]. In addition, the invasion ability of *P*. *gingivalis* ATCC 33277 differs distinctly according to human cell type, such as HeLa cells, Ca9-22 cells, HTR-8 cells, osteoblast, and aortic and heart endothelial cells [17, 25, 33, 37, 38], suggesting that the efficiency of bacterial invasion may be affected by the physiology and differentiation status of the host cell. Additionally, a time course analysis revealed that the invasion efficacy of *P*. *gulae* (Fig 2B) was similar to that previously shown for *P*. *gingivalis* [13]. In contrast, it has been demonstrated that *S*. *aureus* [30] and *Y*. *pestis* [33] require more than 90 min to complete the invasion. These results suggest that the optimal MOI and time course for bacterial invasion differs among bacterial species. Interestingly, another report showed that the optimal MOI and time course differed among members of genus *Burkholderia* [39]. Our results indicate that the conditions for invasion are not different among *Porphyromonas* genus members, though this issue requires additional study.

Several pathogens express a surface protein that binds to cellular receptors, leading to engulfment of the bacterium by cells through cytoskeletal rearrangement and membrane extension [40]. In the present study, SEM and TEM analyses confirmed that the bacteria were surrounded by such membrane protrusions (Figs 6C, 6D, 7C and 7D). A previous report noted that the actin cytoskeleton undergoes a dynamic process of assembly and disassembly during cell crawling, which regulates protrusion formation and contractile filament organization [41]. Treatment of gingival epithelial cells with cytochalasin D blocked *P*. *gulae* adhesion and invasion, while inhibition of microtubule polymerization had no effect (Table 1). Microtubules undergo polarization during migration, though their polarization facilitates trafficking of several molecules at the front to promote protrusion and focal contacts [41]. We found that *P*. *gulae* invasion was blocked by staurosporine, a wide spectrum inhibitor of protein kinase C, though it was not possible to identify the signaling pathways under the present experimental conditions. Staurosporine blocks invasion by some pathogens, including *Y*. *enterocolitica* and *Y*. *pseudotuberculosis*, as well as Shiga toxin-producing *Escherichia coli* [42, 43]. Also, PKC regulates the actin cytoskeleton in a wide range of cell types [44]. Thus, our results suggest that the actin cytoskeleton rather than microtubules may be involved in bacterial adhesion and invasion.

Cycloheximide, an inhibitor of mammalian protein synthesis, did not prevent *P*. *gulae* invasion of gingival epithelial cells (Table 1), as previously reported for *P*. *gingivalis* [13]. Inhibition of adhesion and invasion by azide, a metabolic inhibitor of eucaryotic and procaryotic cells, revealed that *P*. *gulae* and epithelial cell have metabolic requirements related to adhesion and invasion, and also suggest that the energy metabolism of eucaryotic and procaryotic cells is necessary for *P*. *gulae* adhesion and invasion, while *de novo* protein synthesis is not likely required. In contrast, bacterial DNA, RNA, and protein are required for enhancement of *P*. *gulae* adhesion to and invasion of gingival epithelial cells. *P*. *gulae* organisms express several different virulence factors including fimbriae, as well as arginine- and lysine-specific proteinases [9, 45]. A role for *P*. *gulae* proteases is suggested by results showing a protease inhibitor completely inhibited adhesion and invasion (Table 1). Thus, bacterial proteases seem to be involved in the initial attachment of *P*. *gulae* to gingival epithelial cells.

Integrins are receptors of several different pathogens for adhesion to and invasion of host cells [12]. Binding of fibronectin to several bacterial surface proteins through integrin α5β1 has been demonstrated for adhesion and invasion of various bacteria, such as *Haemophilus influenza*, *S*. *aureus*, and *S*. *pseudintermedius* [46, 47]. We found that bacterial adhesion and invasion were significantly inhibited by anti-integrin α5β1 antibodies, whereas anti-integrin αVβ3 antibodies showed negligible effects (5B and C). These findings suggest that integrin α5β1 is a key molecule involved in *P*. *gulae* adhesion and invasion. However, it is notable that binding of *P*. *gulae* to gingival epithelial cells was not completely inhibited by addition of the anti-integrin α5β1 antibody, suggesting an unknown receptor of *P*. *gulae* present in gingivalis epithelial cells.

Invasion of the *P*. *gingivalis* type strain was previously shown to be inhibited by serum, while that of low-passage clinical isolates of *P*. *gingivalis* was not [13]. Moreover, serum has been reported to inhibit adhesion and invasion of *N*. *meningitides*, *Y*. *pestis*, and *S*. *gordonii* [48–50]. Human serum has been shown to induce bacterial aggregation and diminish adhesion and invasion to human cells [50]. In the present study, adhesion and invasion of *P*. *gulae* were inhibited by serum but not plasma (Tables 2 and 3), suggesting that *P*. *gulae* may form aggregates in human serum. These results indicate that adhesion and invasion are controlled by serum components after bacterial infection, thus GCF may have effects on adhesion to and invasion of gingival epithelial cells by *P*. *gulae*.

In conclusion, *P*. *gulae* has an ability to invade human gingival epithelial cells, though invasive efficiency seems to be dependent on *fimA* type. Furthermore, our results suggest that the invasion machinery utilized by *P*. *gulae* may be similar to that of *P*. *gingivalis*. *P*. *gulae* invasion may contribute to the pathogenesis of periodontitis.

## Supporting information

S1 Fig Effects of antibiotics on *P*. *gulae* growth. *P*. *gulae* strains (ATCC 51700, D040, and D049) were incubated with with/without ampicillin (200 μg/ml) and gentamicin (300 μg/ml) for 1 h. After incubation, the strains were anaerobically grown at 37°C for 4 days.
(TIF)

S2 Fig Effects of solvents and inhibitors on Ca9-22 morphology and proliferation. (A) Light microscopy images showing morphology of Ca9-22 cells treated with solvents and inhibitors for 24 h. Control (D049) cells were untreated. (B) Ca9-22 cell proliferation was determined using tetrazolium following treatment with/without solvents and inhibitors for 24 h. Data are expressed as relative to the ratio of treated/untreated and shown as the mean ± SD of three independent experiments. The results were analyzed with a *t* test.
(TIF)

S3 Fig Effects of solvents and inhibitors on *P*. *gulae* growth. All potential inhibitors were examined for any toxic effects on *P*. *gulae*, as determined by counting of viable cells and found to have no adverse effects on viability at the concentrations used. Ethanol, ethyl acetate, DMSO, methanol, and NaOH, used as solvents, were tested at the appropriate concentrations and found to produce no reduction in *P*. *gulae* numbers. Data are expressed as relative to the ratio of treated/untreated and shown as the mean ± SD of three independent experiments. The results were analyzed with a *t* test.
(TIF)

S1 File Summarized values of graph and tables. (XLSX)
