# Supplementary figures and images for: Adhesion and invasion of gingival epithelial cells by Porphyromonas gulae
Source: PLoS One. 2019 Mar 14;14(3):e0213309. doi: 10.1371/journal.pone.0213309 (PMC6417775; doi:10.1371/journal.pone.0213309)

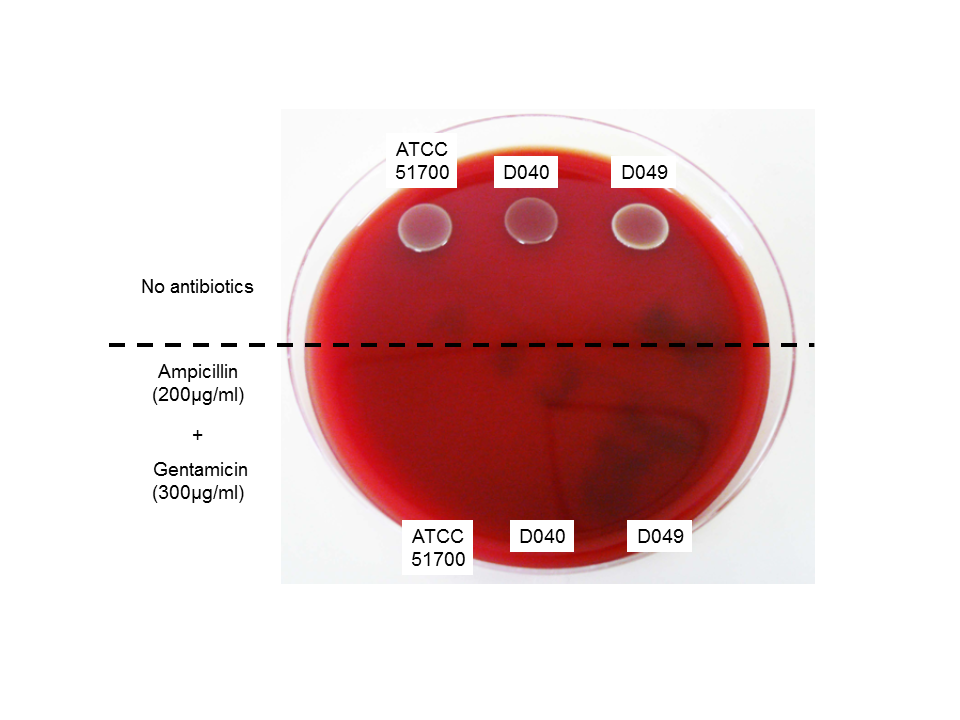

Supplement: S1 Fig — P. gulae strains (ATCC 51700, D040, and D049) were incubated with with/without ampicillin (200 μg/ml) and gentamicin (300 μg/ml) for 1 h. After incubation, the strains were anaerobically grown at 37°C for 4 days. (TIF) [file pone.0213309.s001.tif]

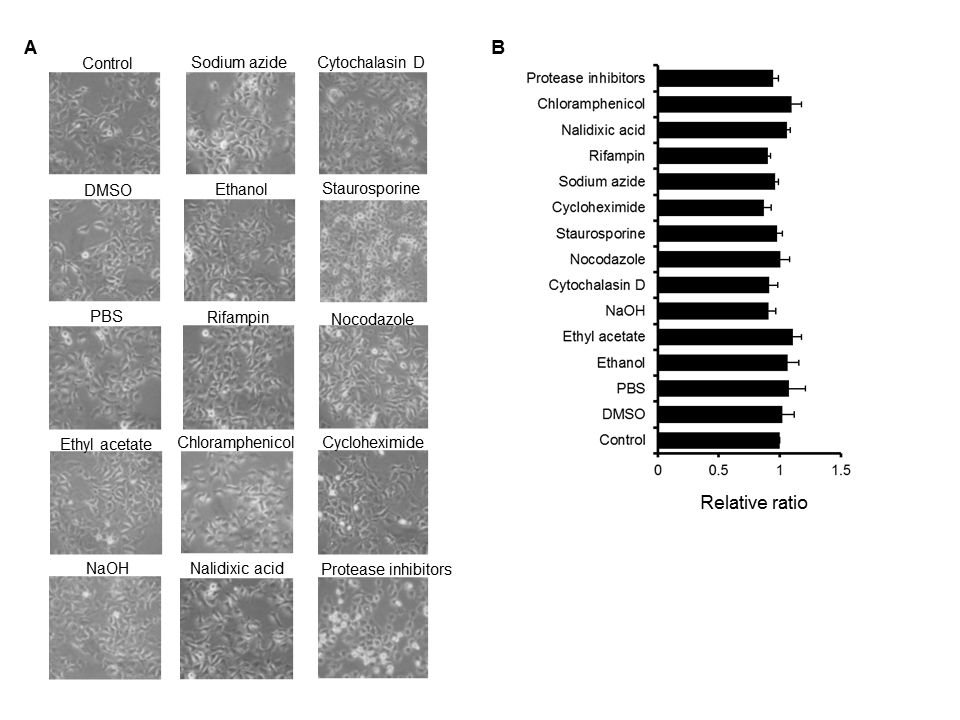

Supplement: S2 Fig — (A) Light microscopy images showing morphology of Ca9-22 cells treated with solvents and inhibitors for 24 h. Control (D049) cells were untreated. (B) Ca9-22 cell proliferation was determined using tetrazolium following treatment with/without solvents and inhibitors for 24 h. Data are expressed as relative to the ratio of treated/untreated and shown as the mean ± SD of three independent experiments. The results were analyzed with a t test. (TIF) [file pone.0213309.s002.tif]

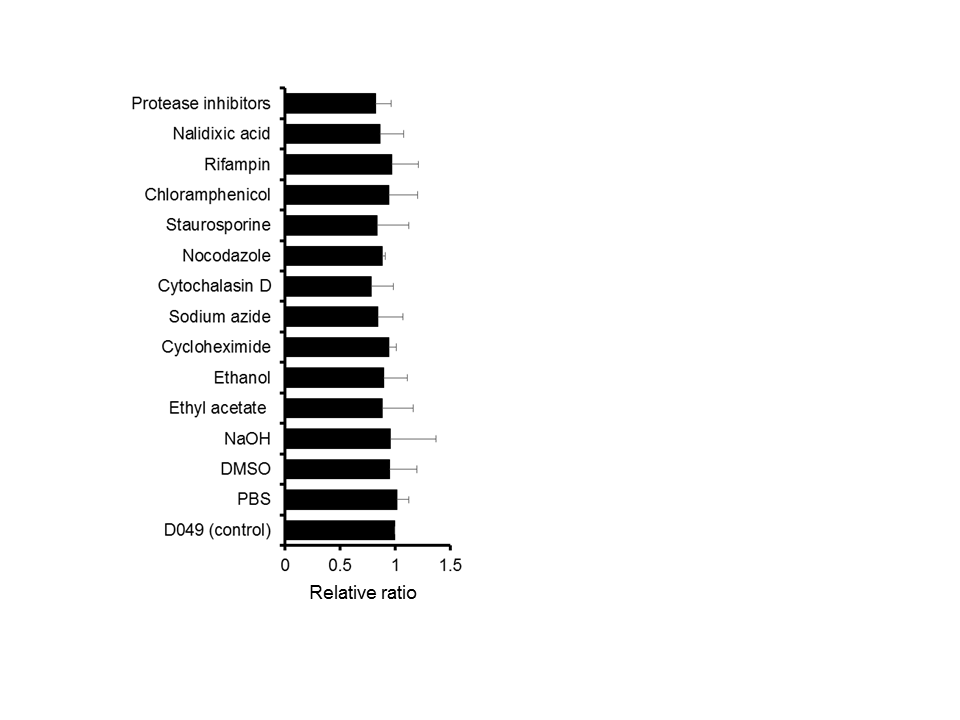

Supplement: S3 Fig — All potential inhibitors were examined for any toxic effects on P. gulae, as determined by counting of viable cells and found to have no adverse effects on viability at the concentrations used. Ethanol, ethyl acetate, DMSO, methanol, and NaOH, used as solvents, were tested at the appropriate concentrations and found to produce no reduction in P. gulae numbers. Data are expressed as relative to the ratio of treated/untreated and shown as the mean ± SD of three independent experiments. The results were analyzed with a t test. (TIF) [file pone.0213309.s003.tif]
